# Data mining and model-predicting a global disease reservoir for low-pathogenic Avian Influenza (A) in the wider pacific rim using big data sets

**DOI:** 10.1038/s41598-020-73664-2

**Published:** 2020-10-08

**Authors:** Marina Gulyaeva, Falk Huettmann, Alexander Shestopalov, Masatoshi Okamatsu, Keita Matsuno, Duc-Huy Chu, Yoshihiro Sakoda, Alexandra Glushchenko, Elaina Milton, Eric Bortz

**Affiliations:** 1 Novosibirsk State University, Novosibirsk, Russia; 2 Federal Research Center of Fundamental and Translational Medicine, Novosibirsk, Russia; 3 EWHALE Lab, Institute of Arctic Biology, Biology and Wildlife Department, University of Alaska Fairbanks (UAF), Fairbanks, USA; 4 Laboratory of Microbiology, Faculty of Veterinary Medicine, Hokkaido University, Sapporo, Hokkaido Japan; 5 Global Station for Zoonosis Control, Global Institute for Collaborative Research and Education (GI-CoRE), Hokkaido University, Sapporo, Hokkaido Japan; 6 Department of Animal Health, Ministry of Agriculture and Rural Development, Ha Noi, Viet Nam; 7 University of Alaska Anchorage (UAA), Anchorage, USA

**Keywords:** Biological techniques, Biotechnology, Computational biology and bioinformatics, Ecology, Microbiology, Zoology, Biogeochemistry, Climate sciences, Ecology, Environmental sciences, Environmental social sciences, Natural hazards, Diseases, Health care, Medical research, Risk factors, Mathematics and computing

## Abstract

Avian Influenza (AI) is a complex but still poorly understood disease; specifically when it comes to reservoirs, co-infections, connectedness and wider landscape perspectives. Low pathogenic (Low-path LP) AI in chickens caused by less virulent strains of AI viruses (AIVs)—when compared with highly pathogenic AIVs (HPAIVs)—are not even well-described yet or known how they contribute to wider AI and immune system issues. Co-circulation of LPAIVs with HPAIVs suggests their interactions in their ecological aspects. Here we show for the Pacific Rim an international approach how to data mine and model-predict LP AI and its ecological niche with machine learning and open access data sets and geographic information systems (GIS) on a 5 km pixel size for best-possible inference. This is based on the best-available data on the issue (~ 40,827 records of lab-analyzed field data from Japan, Russia, Vietnam, Mongolia, Alaska and Influenza Research Database (IRD) and U.S. Department of Agriculture (USDA) database sets, as well as 19 GIS data layers). We sampled 157 hosts and 110 low-path AIVs with 32 species as drivers. The prevalence across low-path AIV subtypes is dominated by Muscovy ducks, Mallards, Whistling Swans and gulls also emphasizing industrial impacts for the human-dominated wildlife contact zone. This investigation sets a good precedent for the study of reservoirs, big data mining, predictions and subsequent outbreaks of HPAI and other pandemics.

## Introduction

Influenza A virus infections are a significant problem affecting the health of wild and domestic animals and public health^1^. The genetic diversity of avian influenza viruses (AIVs) is assumed to be maintained by their circulation in wild aquatic bird populations (see^2–6^ for Pacific Rim region). Avian influenza (AI) is a complex but poorly understood disease which is based on many strains. Some of those are not fully described and are highly pathogenic (hi-path HP, as defined in chicken ). The majority of them is classified as lower pathogeny (low-path LP); those are still underestimated, insufficiently studied and little surveyed even. It has been suggested, but poorly studied, that those AI strains actually co-occur and interact. The prevalence of AI viruses in wild birds varies greatly by species, age, season and geographical location. While species surveying is unequal the highest known prevalence of the 16 haemagglutinin (H1—H16) and nine neuraminidase (N1—N9) subtypes is observed in birds belonging to the Anseriformes and Charadriiformes orders^7,8^. Due to its virulence, public focus, main research attention and subsequent funding sits on high-path AI, whereas the ecologically more relevant low-path AI and its contributions are widely ignored, certainly understudied and consequently not so well managed.

However, the rapid and unpredictable evolution of AI viruses leads to the emergence of new influenza virus strains and subtype combinations, which potentially point towards a global pandemic^3,4,8^. Outbreaks of AI virus infections are known to have serious consequences for animal health and may result in major economic losses for the poultry industry^9^ including product mis-trust, fear, massive financial loss, trade interruption and food insecurity. It’s probably not helpful, and arguably quite dangerous to ignore LP AI in this discussion as it is likely a major stepping stone for any so-called HPAI and pandemic. This is even more important given that co-occurrences of diseases in vectors are likely.

There are well-known landscape hotspots of HPAI^9^, and likely those link with LPAI occurrences and movements as the underlying pool (reservoir). Those AI patterns are increasingly geo-referenced and tracked for origins, nations, and for continents (^6^e.g. https://www.fludb.org/, see^9^ for application), but wider international and cross-continental linkages are hardly coordinated nor well known or studied yet. Since hi-path AI usually comes from areas and hotspots with abundant low-path AI likely it forms a resilient reservoir. But those AI reservoirs and consistent hotspots are also not well identified or studied nor is it understood how they behave over time and seasons (see^9^ for polar breeding seasonalities).

**Figure 1:**
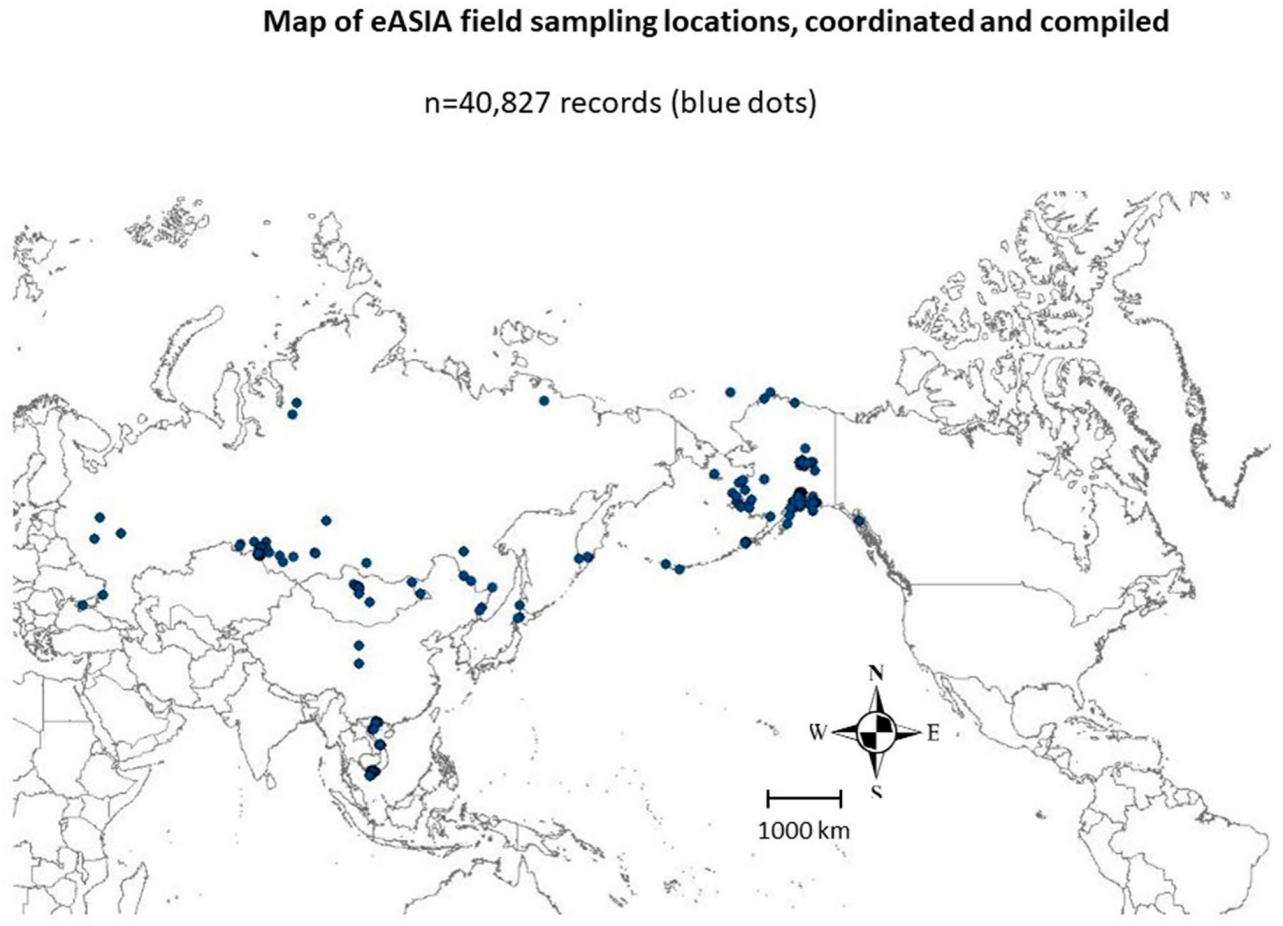
Study area of eASIA project and sampling sites in the Pacific Rim.

To get closer to such type of questions, here we focus on the northern Pacific Rim, a region between North America and Asia, namely Alaska, Russia, Japan and Vietnam (Fig. 1; see^2,9^ for an application). This region is known to be connected through various animal migration patterns (birds^2^ and^10^, marine mammals, mammals, fish and sea turtles), as well as climate regimes. Using the ‘best available’ scientific information on AI for those nations, we then try to obtain alternatively validated AI samples to draw generalizable inferences explicit in space and time.

## Methods

### Study area

The study area consists of the wider northern Pacific Rim area which is known to be an exchange frontier between diseases and cultures (Fig. 1^2,9^). We followed methods outlined in^5,11,12^ and specifically^13^ drawing inference from predictions.

**Figure 2:**
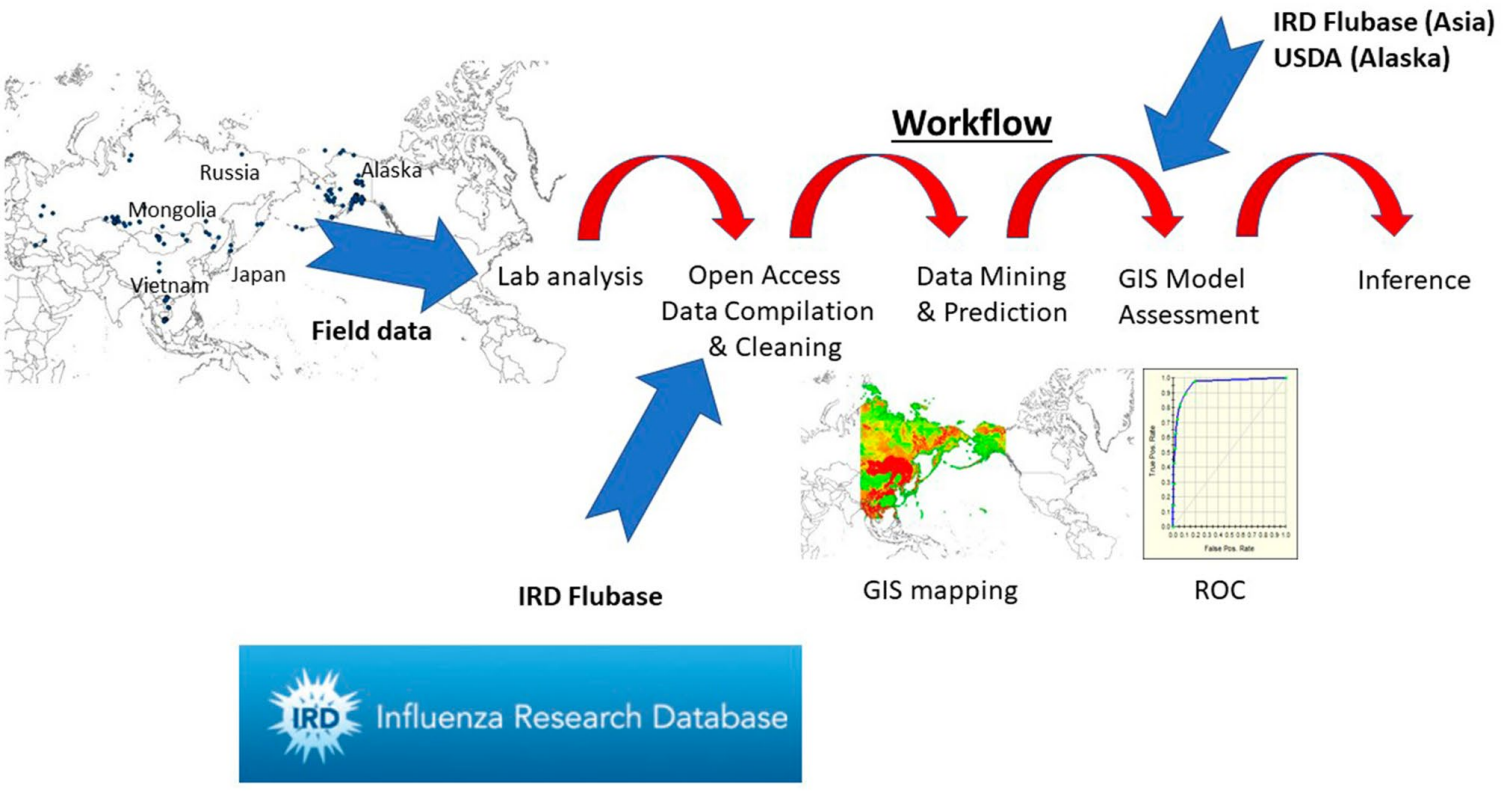
Workflow of this study to obtain best-available AI data and to data mine and predict them with machine learning in a geographic information system (GIS) for best-possible predictions and inference for the Pacific Rim study area (IRD = Influenza Research Database; USDA = U.S. Department of Agriculture); for more details, model specifications etc. see manuscript text.

The conducted international landscape investigation in this study area is described in a research workflow (Fig. 2), and it mainly consists of different steps: field work, open access data compilation, data cleaning and lab work, GIS mapping, data mining and prediction, reflection and inference, as further described below (for more clarifications or questions please contact authors).

### Field work

As part of the eASIA program the field sampling of AI was conducted in Russia and Japan primarily during the fall (August) 2016, 2017 and 2018. Fall is a season when birds finished breeding and started to migrate southwards to their wintering sites. Birds are known during that time to disperse relatively slowly along flyways^10,12,14,15^. Traditionally, this time period has the highest known prevalence of virus, thus far^9^ In Vietnam, the surveillance targeting domestic birds was conducted in summers and falls. Together with all eASIA participants, we extracted data from an agreeable compatible workflow and protocol that allowed for geo-referenced and time-referenced AI samples in the field. Hunters were not directly involved in the study (see permits for bird specimen details). In Russia, following their lab method protocol and according to standard procedures^16,17^ it resulted in 52 samples (10 LPAI presences) from years 2016 and 2017 with 13 unique locations. In Japan, their respective lab method protocol was followed (details in^18^) resulting in 203 samples from years 2016 and 2017 based on 5 unique locations. In Vietnam, the lab method protocol of Japan was followed (details in^19^) resulting in 1,182 samples (951 LPAI presences) from years 2016 and 2017 based on 102 unique locations. Finally, we were also able to obtain 407 samples (395 LPAI presences) for Mongolia for 27 unique locations, also following the protocol from Japan. Alaska was not part of field campaign but had data available through the IRD ‘flu’ database (see details below).

All field data were compiled into one eASIA database for further analysis (Appendix 1), namely to carry out data mining, model-training and subsequent predictions with machine learning and geographic information system (GIS; details in^9,10^).

### Compilations of open access AI data

To reach across the Pacific Rim for a wider and more robust inference, and to make a connection with North America and other available data, further AI data from Alaska were obtained from the IRD database online (https://www.fludb.org/brc/home.spg? Decorator = influenza). This resulted in 38,517 samples (448 low-path AI presences) from 1,175 unique locations. We then queried all these data for low-path AI strains which resulted in 110 strains and 40,837 samples from 157 host species entries that we used for this study (see Appendix 2 for details). To our knowledge, that is the biggest and most diverse AI database ever compiled and analysed for the Pacific Rim (see Herrick et al. 2013 for a first initial model and using all of AI).

### Data mining of low-path AI

We queried the obtained data for the number of low-path AI strains, host species distribution, proportion of host species carrying a specific low-path AI strain, and prevalence.

### Compilations of open access GIS data layers for the study area

GIS layers are used as predictors for model-predictions in the study area. Here we used 19 global GIS layers available from earlier research (Sriram and Huettmann unpublished https://www.earth-syst-sci-data-discuss.net/essd-2016-65/; Table 1). For polygon outlines we used data with our ArcGIS UAF campus license (FH). All GIS data layers were displayed for the study area as a Mercator projection using WGS84, decimal degrees coordinates (latitude and longitude) with a precision of 6 decimals (GPS and GIS, a real world precision of 5 decimals).

**Table 1:** List of GIS Predictors used in this study to data mine and predict low path (LP) Avian Influenza (AI) *

Predictor Number	Predictor Group	Predictor name	Meaning
1	Species	Host species	Species that was caught and samples for AI assessment. This predictor is an attribute of the field-based AI lab data and used in the data mining but it’s not GIS-based and not used in creating the GIS map predictions
2	Landscape classification	Koeppen–Geiger	A widely-used landcover scheme
3		GLC2000	A modern scheme of landcover classes
4		NPD	NPD
5	Livestock	Poultry density	Map of density of poultry per pixel
6		Pig density	Map of density of chicken per pixel
7	Precipitation	March	Rainfall in March
8		June	Rainfall in June
9		September	Rainfall in September
10		December	Rainfall in December
11	Temperature	March	Temperature in March
12		June	Temperature in June
13		September	Temperature in September
14		December	Temperature in December
15	Proximity	Proximity to roads	Road closeness
16		Proximity to coastline	Coastal or not
17	Cyclone	Cyclone	Cyclone occurs in that area, or not
18	Topography	Altitude	Altitude above sea level
19		Slope	Slope in degrees

* Source and details: Except for host species those data come from Sriram and Huettmann (unpublished; https://essd.copernicus.org/preprints/essd-2016-65/).

### GIS mapping and data processing

We used commercial and open source GIS softwares (ArcGIS, QGIS) to operate, map and overlay all data. We imported the AI Data from ASCII table (MS Excel) into a shapefile layer of AI, and overlaid them with 19 environmental GIS layers we had available from compiled global data sets. This resulted into a data cube that is analyzed with data mining and for modeling and predictions.

### Modeling and predictions

The resulting data cube was imported into SPM 8.2 (https://www.minitab.com/en-us/products/spm/) and then modeled and predicted. We ran a stochastic grading boosting (TreeNet) algorithm for best-possible predictions and inference (^20^see also^9,10,12,21^; for an R implementation see^22^). As outlined in^9,12,21^ we started with default settings for this powerful software as they are known to achieve best inference, as taken from the predictive performance^13^. Models then used 6 Maximum nodes per tree, 10 Cases as a Terminal Node Minimum, 200 trees to converge, a balanced class weight and a ten-fold cross-validation (a repeated 90% training vs 10% testing setting) optimizing on the ROC. To avoid overfitting we used an auto learn rate and a 50% subsampling. The resulting tree model was stored as a grove and applied to an equally-spaced lattice of the predictors (excluding species information). The maps were presented in GIS with a resolution of a 5 km pixel size (Appendix 3).

### Model assessment data

We were able to obtain two alternative data set on AI for an assessment of our predictions. The Influenza Research Database (IRD) has an Asian subset (n = 28,205 and 19,405) comparable to our work, and which was used to confront our predictions for the study area.

Although the U.S. Department of Agriculture (USDA) has a U.S-wide AI survey data set (3,589 for Alaska), it actually lacks geo-referencing with coordinates (just done by counties etc.) and just includes H5, H7 Avian Flu columns; presumably done trying to protect the industry. We still used this best-available alternative data set for further assessment of the model predictions.

### Ethics statement

For this eASIA project oropharyngeal and cloacal samples in Russia were collected according to the “Federal Law on Hunting and Sharing of Hunting Resources of Russian Federation # 209-ФЗ” and with the permissions of local governments in hunting regions during each hunting seasons. Hunted birds were provided for sampling by licensed hunters to our group during expeditions.

Fecal samples in Japan were collected with the permission of the municipality managing the sampling areas and Hokkaido University. Fecal samples in Mongolia were collected with the permission of the State Central Veterinary Laboratory, Mongolia. These samples were transferred to Japan under the permissions of the Animal Quarantine Service, Japan (27douken560-2, 28douken563-6, 29douken 683–2). Swab samples in Vietnam were collected with the permission of the Department of Animal Health, Vietnam. These samples were transferred to Japan under the permissions of the Animal Quarantine Service, Japan (27douken560-3, 28douken563-1, 28douken563-4, 28douken563-5, 29douken683-3, 29douken683-4).

Data reported in the Influenza Research Database (IRD) were from samples obtained and submitted under NIH-funded avian influenza surveillance collection efforts (CEIRS) and are publicly available at: www.fludb.org . This work was supported in part by a National Institute of Allergy and Infectious Disease Centers of Excellence in Influenza Research and Surveillance (CEIRS) award, Contract HHSN272201400008C (to Eric Bortz).

For Alaska USDA data, wild bird samples primarily came from hunter-killed waterfowl, with voluntary participation from hunters. These sampling activities were covered under US Fish and Wildlife Service Federal Permit MB124992-0.

## Results

### Data compilation

**Figure 3:**
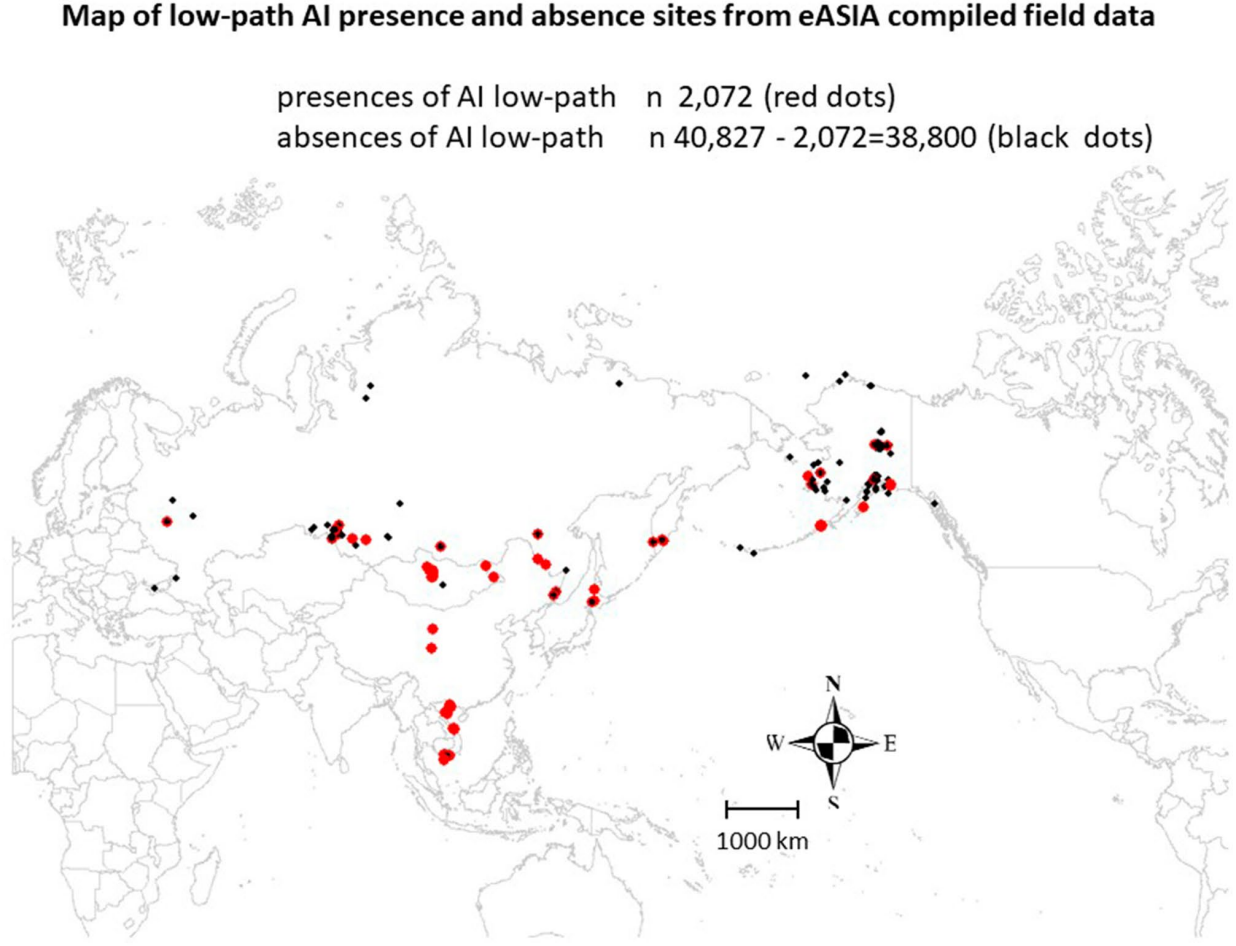
Map of low-path AI data, presence and absence data distribution.

We were able to present the best-available data set on low-path AI—presence/absence—for the Pacific Rim (Fig. 3). We documented this dataset with ISO-compliant metadata (Appendix 1) in an Open Access data sharing framework for the global audience. In addition, we were able to obtain Influenza Research Database (IRD) Asia data as well as the U.S. Department of Agriculture (USDA) Alaska database on Avian Influenza. To our knowledge, there is no better data set for this topic available thus far.

### General AI query and analysis

This is one of the first concerted analyses of low-path AI ever undertaken, also including standardized and shared AI lab work. While the species and study area are widely undersampled, our findings show app. 110 strains of low-path AI, distributed over many bird species. However, of the c. 183 hosts sampled for AI, only 32 carried identifiable low-path AI (details shown in Appendix). Of those species, only a few co-occur, and likely migrate, between the shores of the Pacific Rim in the study area (^6^). Almost all of those species, and especially those with a high prevalence, are from ducks, gulls, and a few shorebirds. The highest prevalence was found with ‘ducks’, chicken, and human-associated species like Muscovy duck, whistling swan, mallards and gulls, for instance. As one of the most abundant species in the study area (^14^, see^9,11^ for an example) passerines were consequently widely undersampled but thus far reported almost no low-path AI. Our study overall did not differentiate between types of AI sampling but most relied on feces. We therefore cover minimum estimates in space and time, for hosts and for low path AI still.

### Prevalence and keystone species

Table 2 shows species with the highest sample sizes and their outcome of low-AI strains (cut-off > 0.2%). The highest prevalences are found for duck and chicken samples (species of tufted duck and whistling swan just carry very low sample sizes and might be considered positive outliers lacking power). Muscovy duck and mallard, as well as environmental samples, should also be considered. All other samples, wild birds, carry relatively low AI subsamples but do occur in the wider reservoir.

**Table 2:** Prevalences of host species for low-path AI strains from the compiled AI dataset.

Host species	AI Samples	Presences of low-path AI	Proportion of sampled species in %
Tufted duck	1	1	100.00
Whistling swan	8	8	100.00
Chicken	488	450	92.21
Duck *	1,120	1015	90.63
Emperor Goose	79	18	22.78
Muscovy duck	103	18	17.48
Environment	259	25	9.65
Mallard	4,338	159	3.67
Green-winged teal	1,448	47	3.25
Pintail	5,166	136	2.63
Ring-necked duck	85	2	2.35
Shoveler	1,514	34	2.25
Gadwall	74	1	1.35
Cackling goose	122	1	0.82
Glaucous-winged gull	3,490	26	0.74
Greater white-fronted goose	527	2	0.38
Sandpiper	644	2	0.31
American Wigeon	1,616	4	0.25
Unidentified Larus gull	422	1	0.24

* Duck undefined usually refers to Mallard or Muscovy Duck.

**Figure 4:**
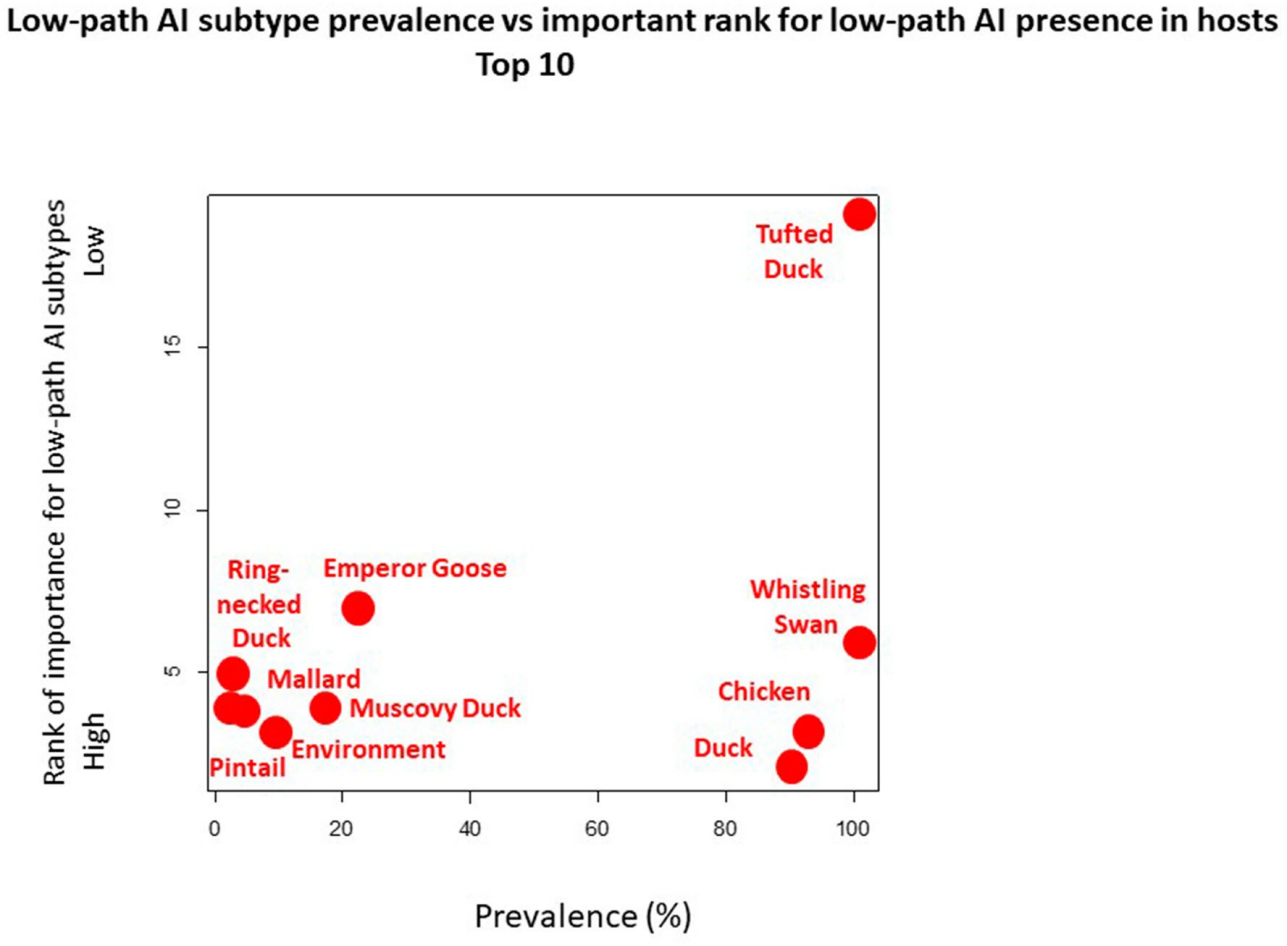
Figure showing relationship between prevalence and importance rank (contribution) to the top 10 low-path AI strains (data shown in Table 2).

**Figure 5:**
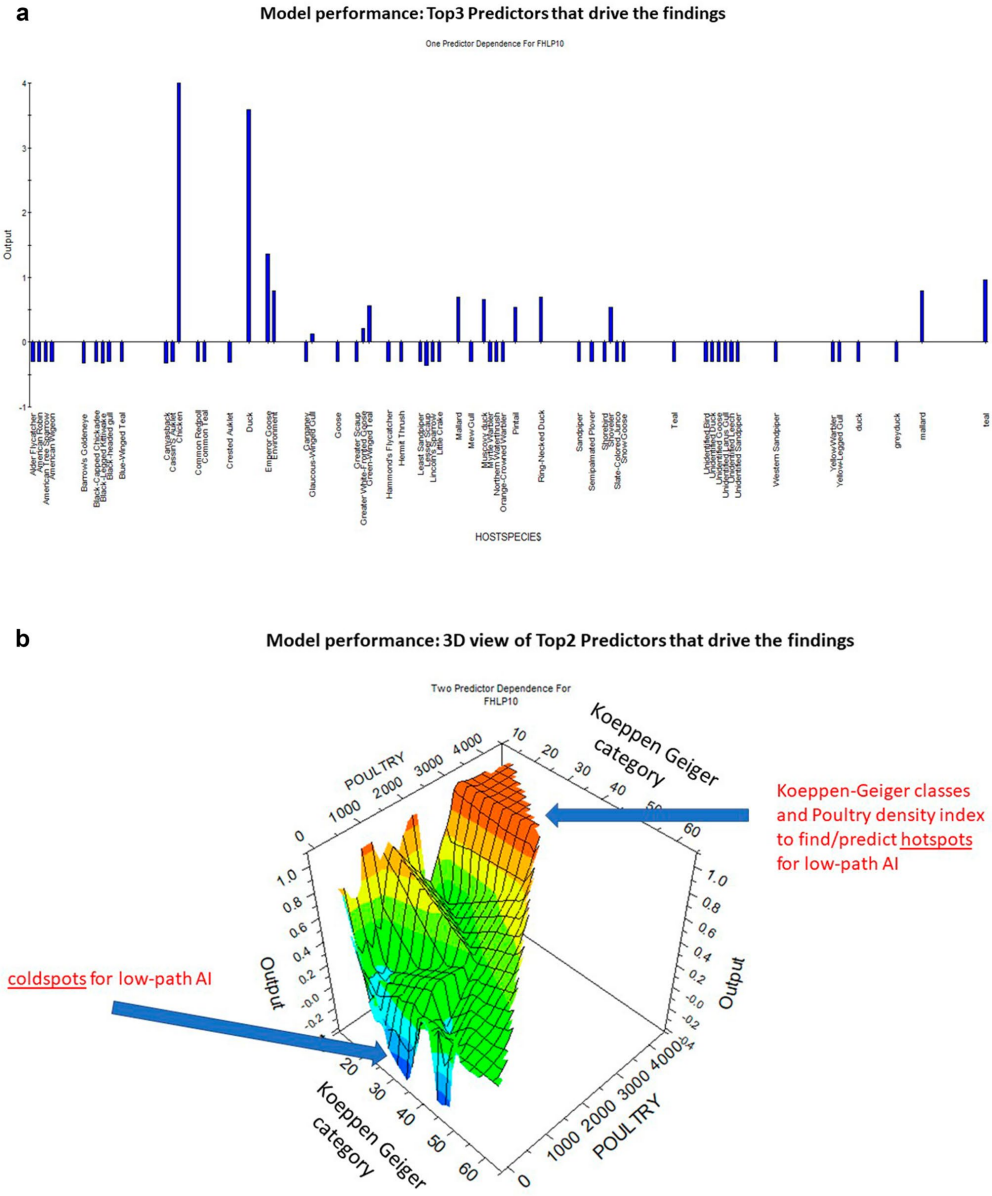
Partial dependence plots of the top 3 predictors a) host species for data mining, and b) showing a 3-dimensional partial dependence plot for predictions (Koeppen Geiger classification and poultry density index).

The Appendix shows the most dominant low-path AI strains and with their associated host diversity and major contributing hosts. Low-path AI co-occurs in several species and might be found as a community. A low path AI strain is found in average in over 7 different host species (for the Top 20 hosts). Figure 4 summarizes the relationship between prevalence and contribution rank for the major low-path AI strains. It finds that chicken, ducks and human-associated waterfowl species like Muscovy duck and mallards, as well as Larid gulls seem to play a major ecological role for low-path AI. Figure 5 shows how those species contribute to the model and how location and human factors interact towards low path AI prediction.

### Model-details and predictions

**Figure 6:**
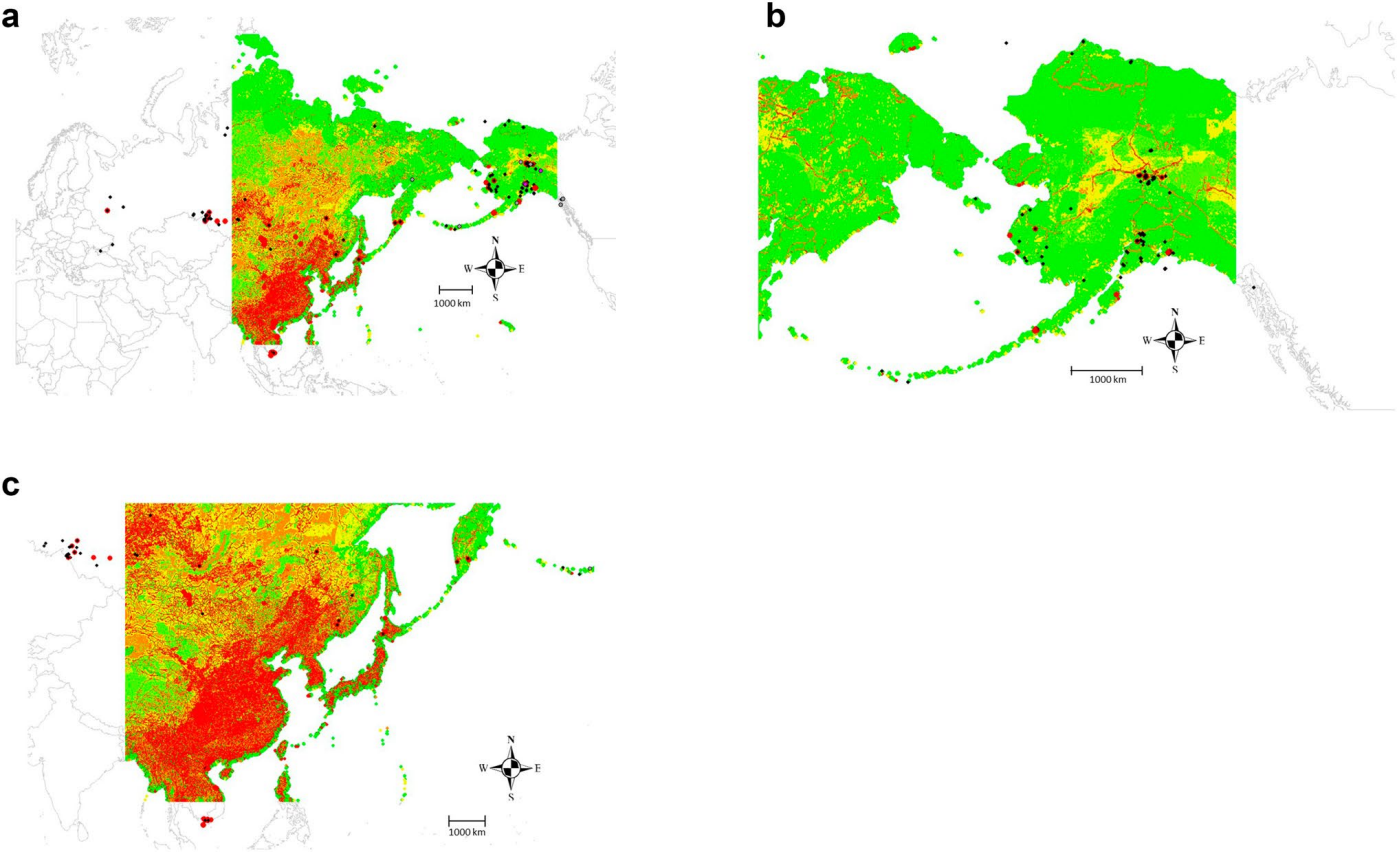
a) Model-predicted surface of low-path AI, b) Alaska zoom-in, c) Asia zoom-in. This map shows a heatmap where predicted presence and absence is shown as a relative index of occurrence (RIO with red = presence, green = absence, and gradient colors in-between).

Our model predictions are the first type of inference for low-path AI and its compiled best-available public data set. We present a model prediction surface in Fig. 6, showing a hotspot in Asia, namely China, coastal Asia, central Siberia and a more mixed-pixel and declining gradient further north. A connecting corridor of low-path AI would be possible between Asia with Alaska across the dateline but is not very dominant.

For predicted coldspots (= absence) they seem to occur in the high arctic and in areas that are less populated or lack urbanization as well as the are not within the immediate coastal zones.

Our model is based on 19 predictors, of which app. 5 are among the most important ones acting in concert (Table 3). We wish to see it interpreted as a multivariate set of predictors in which low-path AI can be predicted well (ROC of over 90%). This set of relevant predictors for low-path AI has a co-occurring scheme. It consist of anthropogenic factors in the tropical Asian landscape such as roads and road proximity, poultry density and landcover types that have a human population and development on a global scale. It shows a direct affiliation with relevant centers of the world’s economic growth.

**Table 3:** Importance ranking of predictors for low-path AI model based on Treenet algorithm (SPM).

Predictor rank of importance	Name of predictor	Percent importance ranking
1	Host species*	100
2	Koeppen geiger classification	24
3	Proximity to known poultry farms	13
4	Mean precipitation June	12
5	Proximity to roads	12
6	GLC2000 landcover	11
7	Slope in degrees	11
8	Proximity to coast	10
9	Proximity to pig farms	9
10	Mean precipitation September	8
11	Mean temperature December	8
12	Altitude	7
13	Nationalpark	7
14	Mean temperature March	6
15	Mean precipitation March	6
16	Mean temperature September	5
17	Mean temperature June	5
18	Mean precipitation December	5
19	Located in cyclone area	3

* An attribute that is associated with AI lab data; it was used for the data mining (not landscape prediction surface as such information is not really available on a landscape scale; see also^10,15^)

The host species makes for the major driver of low-path AI in the Pacific Rim. But arguably, the host species occurrence is eventually determined by the ecological niche, which consists, in a large part, of predictors we used in this model. Those show us a multivariate set of predictors that determine the response of low-path AI (details shown Fig. 5). Beyond the identified Koeppen Geiger classes—namely categories in Western China, the triangle between Mongolia- Russia-China, Southern Japan and Vietnam—individual climate predictors like monthly temperature and precipitation play less of a role for low-path AI and human factors dominate overall.

### Model assessment

**Figure 7:**
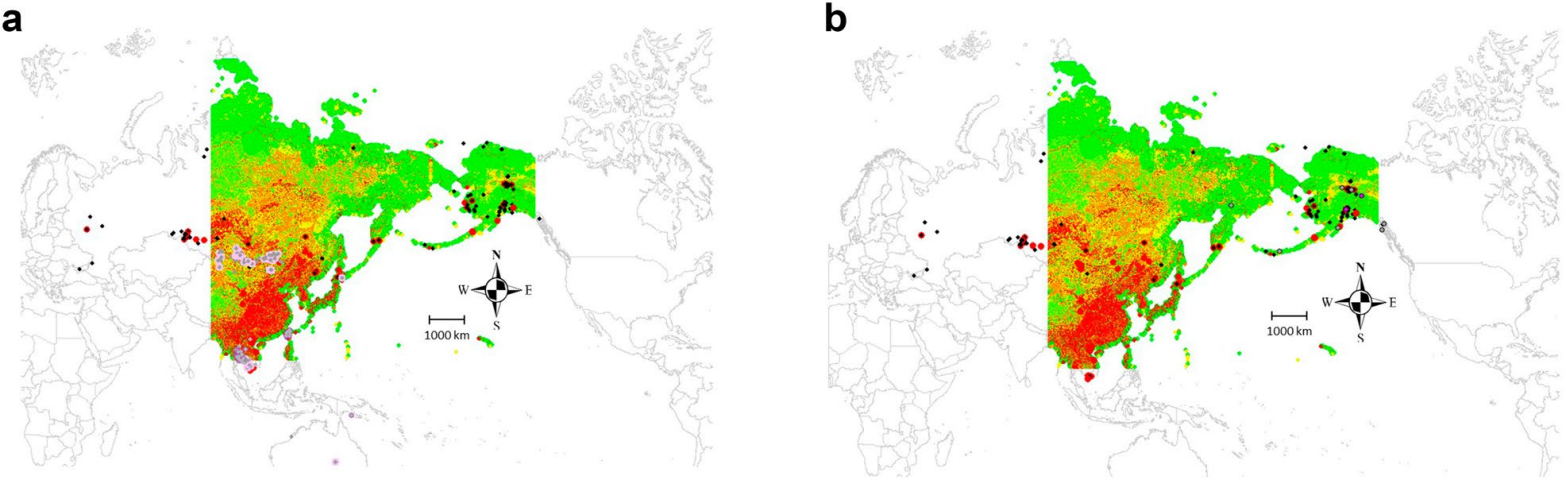
Model assessment and overlay of predicted surface of low-path AI vs alternative AI data a) IRD Asia data, b) USDA Alaska data.

Based on confronting low-path AI predictions for an assessment with alternative data we find a good match with the IRD data for Asia (Fig. 7a). While the second testing data from USDA is not geo-referenced with coordinates but uses counties, and just sampled for AI presence, H5 and H7, it cannot fully be compared. However, while with less evidence, it also shows a general match with our data (Fig. 7b) indicating that LP AI could relate to HP AI even.

## Discussion

One of the fundamental unknowns in the field of influenza biology is a panoramic understanding of the role wild birds play in the global maintenance and spread of influenza A viruses. AI may be perceived as an industrial disease with commercial chicken and ducks playing the major roles and ecological spill-over effects into the wild. A well-known fact is that wild aquatic birds are considered a reservoir host for all low pathogenic avian influenza A viruses. Thus, genes of low path viruses may contribute to the emergence of pandemic viruses responsible for morbidity and mortality in both poultry and humans worldwide. Therefore presenting reservoir locations is important information to identify and treat a potential source of zoonotic AIV (^9,23^).

Here we were able to compile and document the best-available (‘Big Data’) data set for LPAI in the Pacific Rim study area, available as a publically-available GIS layer with ISO-compliant metadata. Further, we were able to create the best-possible publically available prediction of low-path AI for the Pacific Rim using machine learning and open access data. In addition, we were able to obtain and use two alternative low-path AI data sets to confront the model predictions for validity: U.S. IRD Asia and USDA Alaska. It is supposed to be the first ever ‘Big Data’ synthesis analysis across years, nations and data sets for AI done anywhere (compare with^6^ and^9^). This work is based on the coordinating eASIA project for the Pacific Rim allowing for international views of AI and public health perspectives.

Arguably the data mining workflow and international large-scale multi-lab methodology is the first of its kind allowing for Ecological Niche analysis and inference (Fig. 2; see^9^ for generic AI). Our field sampling work is still incomplete on a landscape-scale though and lacks a research design assessment for effectiveness, which is to be improved in subsequent efforts. However, here we set a first and digital baseline to start from, all in Open Access formats to work from further, e.g. filling sampling gaps, pursuing specific research and management questions, and improving and testing model predictions. Further, quality control of AI data is to improved, standardized and assessed also, specifically detection rates in the field and with certified lab protocols.

Although it is one of the largest AI studies ever done, our data are still widely undersampling the species in the vast landscapes^10,15^. We therefore report underestimates. Looking at co-occurrences, we found that app. 32 host species are involved—including the environment- for low-path AI. We also find that low-path AI are found in many hosts, e.g. over 7 species on average for the top 20 low-path AI strains. From the data at hand, one can easily see that human-dominated species such as chicken and duck -including mallards and Muscovy ducks—play a central role for low-path AI. However, the wild species component remains widely undersampled but matters with wider ecological reality to focus on.

Our prediction maps are able to show hotspots in Asia, namely China, coastal Asia, parts of Central Siberia, as well as a connecting ‘flyways’, with a lower proportion in higher latitudes. Similar to findings in Asia, in Alaska, urban centers, roads and river plains seem to host much of the low-path AI in the landscape. Our hotspots are based on the widely proven Ecological Niche analysis concept^9,12^ and the synthesis shows a co-occurrence with areas of globally recognized high human populations, development and subsequent economic growth. There is a concern then that AI can spread and transfer from these regions further, affecting livelihoods, wilderness and mankind worldwide (^9,24,25^). That’s where a focus on more ecological perspectives, connectivity and spill-over effects (‘telecoupling’^26^) provides more progress.

The assessment data indicate that our model predictions are pretty robust. This must not come as a big surprise when knowing the reliability of machine learning modeling methods in space and time (see for instance^9,13^, and^12^ for generic applications and performance).

This study sets a baseline, and it now can be improved further, namely making good use of digital products compiled and created. Further we suggest a focus on holistic/ecological approaches, an increased representative sampling of all species and landscapes (hotspots, coldspots, gradients in space and time), coordinating sampling and public data sharing with other projects and hotspots elsewhere, e.g. in the European Union and with the World Health Organization. Also more assessments should be carried out, and data accuracy and sharing are to be improved, e.g. for Alaska, geo-referencing using quantitative coordinates with 6 decimals and providing AI subtype information all done open access with ISO-compliant metadata.

Here we were able to present a first Big Data low-path AI perspective and to highlight hotspots, coldspots and reservoirs for improved handling, studying, and management of AI in the Pacific Rim and globally. We think this work allows for a template to gain better inference and for better management of low-path AI and AI overall using modern methods.

## Supplementary information

Supplementary information 1.

Supplementary information 2.

Supplementary information 3.

Supplementary information 4.
