# Author Correction: Data mining and model-predicting a global disease reservoir for low-pathogenic Avian Influenza (AI) in the wider Pacific Rim using big data sets

**DOI:** 10.1038/s41598-021-83100-8

**Published:** 2021-02-08

**Authors:** Marina Gulyaeva, Falk Huettmann, Alexander Shestopalov, Masatoshi Okamatsu, Keita Matsuno, Duc‑Huy Chu, Yoshihiro Sakoda, Alexandra Glushchenko, Elaina Milton, Eric Bortz

**Affiliations:** 1 Novosibirsk State University, Novosibirsk, Russia; 2 Federal Research Center of Fundamental and Translational Medicine, Novosibirsk, Russia; 3 EWHALE Lab, Institute of Arctic Biology, Biology and Wildlife Department, University of Alaska Fairbanks (UAF), Fairbanks, USA; 4 Laboratory of Microbiology, Faculty of Veterinary Medicine, Hokkaido University, Sapporo, Hokkaido Japan; 5 Global Station for Zoonosis Control, Global Institute for Collaborative Research and Education (GI‑CoRE), Hokkaido University, Sapporo, Hokkaido Japan; 6 Department of Animal Health, Ministry of Agriculture and Rural Development, Ha Noi, Viet Nam; 7 University of Alaska Anchorage (UAA), Anchorage, USA

Correction to: *Scientific Reports* https://doi.org/10.1038/s41598-020–73664-2, published online 08 October 2020

The original version of this Article contained an error in the title of the paper, where the term “Avian Influenza” was incorrectly abbreviated as “(A)” instead of “(AI)”.

Additionally, the Acknowledgements section was incomplete.

“We are grateful to our eASIA funders; the kind collaboration and efforts are widely acknowledged. Further we acknowledge the contributions of all data providers in IRD, as well as USDA for sharing their coarse non-geo-referenced data. FH acknowledges the kind Salford Predictive Modeler (SPM) -Minitab- software license support, the efficient UAF Writing Center, as well as the great Cup and Porcupine and their support and full recovery during this study. The study was funded by RFBR according to the research project № 18-54-70006. This is EWHALE lab publication # 251.”

now reads:

“We are grateful to our eASIA funders; the kind collaboration and efforts are widely acknowledged. Further we acknowledge the contributions of all data providers in IRD, as well as USDA for sharing their coarse non-geo-referenced data. FH acknowledges the kind Salford Predictive Modeler (SPM) -Minitab- software license support, the efficient UAF Writing Center, as well as the great Cup and Porcupine and their support and full recovery during this study. The study was funded by RFBR according to the research project № 18-54-70006. This is EWHALE lab publication # 251. This work was in part supported by a NIAID CEIRS award (HHSN272201400008C).”

This has now been corrected in the PDF and HTML versions of the Article, and in the accompanying Supplementary information 1 file.

