# Supplementary material for: Data mining and model-predicting a global disease reservoir for low-pathogenic Avian Influenza (A) in the wider pacific rim using big data sets
Source: Sci Rep. 2020 Oct 8;10:16817. doi: 10.1038/s41598-020-73664-2 (PMC7545095; doi:10.1038/s41598-020-73664-2)
Supplement: Supplementary file 1 — Supplementary information 1. [file 41598_2020_73664_MOESM1_ESM.pdf]

Title Page:

Data Mining and Model-predicting a global disease reservoir for low-pathogenic Avian Influenza (A) in the wider Pacific Rim using Big Data sets

Marina Gulyaeva [1,2], Falk Huettmann [3*], Alexander Shestopalov [2], Masatoshi Okamatsu [4], Keita Matsuno [4,5], Duc-Huy Chu [6], Yoshihiro Sakoda [4,5], Alexandra Glushchenko [2], Elaina Milton [7], Eric Bortz [7]

1. Novosibirsk State University, Novosibirsk, Russia

2. Federal Research Center of Fundamental and Translational Medicine, Novosibirsk, Russia

3. EWHALE lab, Inst of Arctic Biology, Biology & Wildlife Dept, University of Alaska Fairbanks

* corresponding author: fhuettmann@alaska.edu

4. Laboratory of Microbiology, Faculty of Veterinary Medicine, Hokkaido University, Sapporo, Hokkaido, Japan.

5. Global Station for Zoonosis Control, Global Institute for Collaborative Research and Education (GI-CoRE), Hokkaido University, Sapporo, Hokkaido, Japan

6. Department of Animal Health, Ministry of Agriculture and Rural Development, Ha Noi, Viet Nam

7. University of Alaska Anchorage

Appendix 1 List of 110 low pathogenic AI strains used for model-prediction

H1N1, H3N8, H4N6, H12N5, H4N8, H4N5, H7N3, H3N1, H10N7, Mixed, H9N2, H3N6, Mixed,H4N6, H7N3,Mixed, H3N8,H4N6, H3N8,H7N3, H10N6, H12N8, H8N4, H10N4, N6, H4N7, H6N2, H6N1, H16N3, N3, H16N3,H13N8,unknown,mixed/H13,Mixed,H13N6, Mixed,Mixed/H5, H11N9, H13N6 H3N7, H3N3, H11N3, HxN7, H13N2, H6N8, mixed/H3, H7N7, H15N4, H4N1, H6N6, H4, H6, H3N2, H11N8, H3N5, H10N8, H1N8, H10N3, H7N6, H10N2, H1N2, H8N6, H10N9, H6N5,H1N3, H4N3, H7N9, H11N6, H11N2, H7N2, H4N2, H12N1, H6N9, H3, H12N2, H8N2, H1N5, H5N6, H9N8, H11N7 ,H9N6, H7N1, H11N5, H13N8

Appendix 2 Raw data list of species and host summaries with AI samples used in this Pacific Rim study (Ranked by Percentage. Details and spellings presented as taken from the databases described in the text). Note: Entries without proper GIS layer referencing were deleted from the subsequent analysis.

Hostspecies	CountOfHostspecies	Positive	Ratio	Percentage
Whistling swan	8	8	1.00	100.00
slaty-backed gull	5	5	1.00	100.00
falcated teal	1	1	1.00	100.00
Scourer	1	1	1.00	100.00
sea-gull	1	1	1.00	100.00
white-headed duck	1	1	1.00	100.00
Chicken	504	452	1.12	89.68
Duck	1141	1017	1.12	89.13
herring gull	8	5	1.60	62.50
gull	14	7	2.00	50.00
wigeon	3	1	3.00	33.33
Emperor Goose	83	18	4.61	21.69
Muscovy Duck	104	19	5.47	18.27
tufted duck	7	1	7.00	14.29
Environment	262	25	10.48	9.54
pochard	21	2	10.50	9.52
Common Goldeneye	16	1	16.00	6.25
Mallard	4406	171	25.77	3.88
coot	27	1	27.00	3.70
Green-Winged Teal	1449	47	30.83	3.24
pintail	5202	146	35.63	2.81
shoveler	1558	42	37.10	2.70
Ring-Necked Duck	85	2	42.50	2.35
Cackling Goose	128	2	64.00	1.56
Gadwall	94	1	94.00	1.06
Greater White-Fronted Goose	534	3	178.00	0.56
Least Sandpiper	1254	5	250.80	0.40
sandpiper	645	2	322.50	0.31
American Wigeon	1616	4	404.00	0.25
Glaucous-Winged Gull	11839	28	422.82	0.24
Unidentified Larus Gull	423	1	423.00	0.24
Lesser Scaup	1430	0	0.00	0.00
Western Sandpiper	1334	0	0.00	0.00
Shorebird	549	0	0.00	0.00
Unidentified Goose	489	0	0.00	0.00
Unidentified Sandpiper	488	0	0.00	0.00
Myrtle Warbler	434	0	0.00	0.00
Lincoln's Sparrow	381	0	0.00	0.00
Unidentified Duck	366	0	0.00	0.00
Unidentified Bird	306	0	0.00	0.00

Dusky Canada Goose	279	0	0.00	0.00
Slate-Colored Junco	255	0	0.00	0.00
Orange-Crowned Warbler	249	0	0.00	0.00
Yellow-Legged Gull	242	0	0.00	0.00
Common Redpoll	191	0	0.00	0.00
teal	178	0	0.00	0.00
Snow Goose	164	0	0.00	0.00
Hammond's Flycatcher	150	0	0.00	0.00
Greater Scaup	140	0	0.00	0.00
Canvasback	114	0	0.00	0.00
Northern Waterthrush	109	0	0.00	0.00
Black-Legged Kittiwake	106	0	0.00	0.00
Swainson's Thrush	88	0	0.00	0.00
Black-Capped Chickadee	86	0	0.00	0.00
American Robin	84	0	0.00	0.00
Yellow Warbler	72	0	0.00	0.00
Leach'S Storm-Petrel	70	0	0.00	0.00
Bufflehead	62	0	0.00	0.00
Common Teal	61	0	0.00	0.00
Unknown	58	0	0.00	0.00
Mew Gull	52	0	0.00	0.00
American Tree Sparrow	38	0	0.00	0.00
Wilson's Warbler	38	0	0.00	0.00
Least Auklet	37	0	0.00	0.00
Garganey	35	0	0.00	0.00
Fork-Tailed Storm Petrel	33	0	0.00	0.00
Savannah Sparrow	29	0	0.00	0.00
Domestic Chicken	27	0	0.00	0.00
Alder Flycatcher	26	0	0.00	0.00
Ruby-Crowned Kinglet	25	0	0.00	0.00
Unidentified Scaup	25	0	0.00	0.00
Common Murre	23	0	0.00	0.00
Canada Goose	21	0	0.00	0.00
Crested Auklet	21	0	0.00	0.00
Fox Sparrow	20	0	0.00	0.00
Gambel's White-Crowned Sparrow	19	0	0.00	0.00
Rusty Blackbird	17	0	0.00	0.00
Blue-Winged Teal	15	0	0.00	0.00
Boreal Chickadee	14	0	0.00	0.00
Hermit Thrush	14	0	0.00	0.00
Horned Puffin	14	0	0.00	0.00
Red Legged Kittiwake	13	0	0.00	0.00
King Eider	12	0	0.00	0.00
Sandhill Crane	12	0	0.00	0.00
Semipalmated Plover	11	0	0.00	0.00

Barrow's Goldeneye	10	0	0.00	0.00
Grey-Cheeked Thrush	10	0	0.00	0.00
Long-Tailed Duck	10	0	0.00	0.00
Brant	9	0	0.00	0.00
Pigeon Guillemot	9	0	0.00	0.00
Harlequin Duck	7	0	0.00	0.00
Swan	7	0	0.00	0.00
Tree Swallow	7	0	0.00	0.00
Bald Eagle	6	0	0.00	0.00
Black-headed gull	6	0	0.00	0.00
Common Raven	6	0	0.00	0.00
Redhead	6	0	0.00	0.00
Tufted Puffin	6	0	0.00	0.00
Blackpoll Warbler	5	0	0.00	0.00
Semipalmated Sandpiper	5	0	0.00	0.00
Solitary Sandpiper	5	0	0.00	0.00
Whooper swan	5	0	0.00	0.00
Wood Thrush	5	0	0.00	0.00
Black Scoter	4	0	0.00	0.00
Golden-Crowned Kinglet	4	0	0.00	0.00
greyduck	4	0	0.00	0.00
Red-Necked Phalarope	4	0	0.00	0.00
shoveller	4	0	0.00	0.00
Townsend's Warbler	4	0	0.00	0.00
Trumpeter Swan	4	0	0.00	0.00
Varied Thrush	4	0	0.00	0.00
Violet-Green Swallow	4	0	0.00	0.00
Cormorant	3	0	0.00	0.00
crow	3	0	0.00	0.00
goldeneye	3	0	0.00	0.00
Northwestern Crow	3	0	0.00	0.00
redpoll	3	0	0.00	0.00
Arctic Tern	2	0	0.00	0.00
Black Oystercatcher	2	0	0.00	0.00
Downy Woodpecker	2	0	0.00	0.00
Eider	2	0	0.00	0.00
Goose	2	0	0.00	0.00
Great black-headed gull	2	0	0.00	0.00
Great Crested Grebe	2	0	0.00	0.00
Grey Phalarope/Red Phalarope	2	0	0.00	0.00
Marbled Murrelet	2	0	0.00	0.00
Rook	2	0	0.00	0.00
Stellers Eider	2	0	0.00	0.00
Stellers Jay	2	0	0.00	0.00
Steppe Gull	2	0	0.00	0.00

turnstone	2	0	0.00	0.00
White-Crowned Sparrow	2	0	0.00	0.00
Duck	1	0	0.00	0.00
/tufted duck	1	0	0.00	0.00
Arctic Warbler	1	0	0.00	0.00
Baikal tea	1	0	0.00	0.00
Baird's Sandpiper	1	0	0.00	0.00
Bank Swallow	1	0	0.00	0.00
Bar-Tailed Godwit	1	0	0.00	0.00
bittern	1	0	0.00	0.00
Black-Billed Magpie	1	0	0.00	0.00
Bonaparte'S Gull	1	0	0.00	0.00
Cassin'S Auklet	1	0	0.00	0.00
Common Loon	1	0	0.00	0.00
Common moorhen	1	0	0.00	0.00
Dark-Eyed Junco	1	0	0.00	0.00
Dunlin	1	0	0.00	0.00
Gadwall Hybrid	1	0	0.00	0.00
Golden-Crowned Sparrow	1	0	0.00	0.00
Great Blue Heron	1	0	0.00	0.00
Great Horned Owl	1	0	0.00	0.00
grebe	1	0	0.00	0.00
Hand	1	0	0.00	0.00
Horned Grebe	1	0	0.00	0.00
lapwing	1	0	0.00	0.00
Little Crake	1	0	0.00	0.00
Long-Billed Dowitcher	1	0	0.00	0.00
Magpie	1	0	0.00	0.00
Mallard X Northern Pintail Hybrid	1	0	0.00	0.00
merganser	1	0	0.00	0.00
Pacific Loon Or Diver	1	0	0.00	0.00
Pacific Seagull	1	0	0.00	0.00
Pectoral Sandpiper	1	0	0.00	0.00
pigeon	1	0	0.00	0.00
Pine Grosbeak	1	0	0.00	0.00
Red-crested Pochard	1	0	0.00	0.00
Rock Pigeon	1	0	0.00	0.00
Sabine's Gull	1	0	0.00	0.00
Scoter	1	0	0.00	0.00
Smew	1	0	0.00	0.00
Spectacled Eider	1	0	0.00	0.00
Storm Petral	1	0	0.00	0.00
Surf Scoter	1	0	0.00	0.00
swine	1	0	0.00	0.00
toadstool	1	0	0.00	0.00

Tristram's Storm Petrel	1	0	0.00	0.00
turtledove	1	0	0.00	0.00
Unidentified Leech	1	0	0.00	0.00
Unknown Goldeneye	1	0	0.00	0.00
Water rail	1	0	0.00	0.00
White-Billed Diver/Yellow-Billed Loon	1	0	0.00	0.00
white-fronted goose	1	0	0.00	0.00
White-Winged Crossbill	1	0	0.00	0.00
White-Winged Scoter	1	0	0.00	0.00
White-Winged Scoter	1	0	0.00	0.00

Appendix 3

GIS layers used for predicted low-path AI in the Pacific Rim

Digital ESRI point pixel file in latitude longitude coordinate system (WGS84; shown in Mercator projection for the Pacific Rim; as per [5.]).

Appendix 3.1 : Data compilation and metadata

a) AI data (field work, compiled data for the Pacific Rim)
b) AI data (IRD, Pacific Rim)
c) AI data USDA (Alaska)

Appendix 3.2: Prediction GIS surface (point lattice with predicted RIO Relative Index of Occurrence). This data can be smoothed and/or is available as a GIS grid surface as well upon request

Index of file names and data content:

BaseMapPacRim3merc.shp	Study area and country boundary (GIS shape file)
PooledCleanbyFH5.shp and csv	Compiled Low Path (LP) Avian Influenza data (GIS shape file and ASCII CSV)
5kmrgntstybxmLNDII2overexportedxy_score1.shp	TreeNet prediction (GIS shape file)
IRDAsiaCountriesAMFH2c.shp	IRD LPAI data used for Model Assessment (Mongolia, Vietnam etc.) (GIS shape file)
IRDAsiaCountriesPZFH2c	IRD LP AI data for Model Assessment (Thailand, Taiwan etc) (GIS shape file)
AI20152017PACHueHAKIFH1.shp	Coarse USDA Alaska data for Model Assessment High Path AI 5&7 (GIS shape file)
