# Supplementary material for: Data mining and model-predicting a global disease reservoir for low-pathogenic Avian Influenza (A) in the wider pacific rim using big data sets
Source: Sci Rep. 2020 Oct 8;10:16817. doi: 10.1038/s41598-020-73664-2 (PMC7545095; doi:10.1038/s41598-020-73664-2)
Supplement: Supplementary file 2 — Supplementary information 2. [file 41598_2020_73664_MOESM2_ESM.html]

Compiled avian influenza (AI) data for the Pacific Rim for the eAsia project (2020)


# Compiled avian influenza (AI) data for the Pacific Rim for the eAsia project (2020)

Metadata also available as

### Metadata:

- Identification\_Information
- Data\_Quality\_Information
- Spatial\_Data\_Organization\_Information
- Spatial\_Reference\_Information
- Entity\_and\_Attribute\_Information
- Distribution\_Information
- Metadata\_Reference\_Information

Identification\_Information:

Citation:

Citation\_Information:

Originator:

Marina Gulyaeva, Falk Huettmann, Alexander Shestopalov, Masatoshi Okamatsu, Keita Matsuno, Duc-Huy Chu , Yoshihiro Sakoda , Elaina Milton, Eric Bortz

Originator:Publication\_Date: 20191104  
Title:

Compiled avian influenza (AI) data for the Pacific Rim for the eAsia project (2020)

Edition: 1  
Geospatial\_Data\_Presentation\_Form: database  
Series\_Information:

Series\_Name: 1  
Issue\_Identification: 1

Publication\_Information:

Publication\_Place: University of Alaska Fairbanks  
Publisher: University of Alaska

Other\_Citation\_Details: NA  
Online\_Linkage: NA

Description:

Abstract:

This dataset is a compilation for Avian Influenza data in the Pacific Rim for the eASIA project.
It covers over 40,000 low-path (LP) AI geo-referenced samples for Japan, Vietnam, Mongolia, Russia and Alaska, US.
Data come from coordinated field work campaigns, as well as the IRD Flubase; a value-added product in GIS (shapefile and its Open Access derivatives). Overall, over 100 strains are presented from over 150 host species, including environmental samples. These are training data for model prediction of the low-path AI reservoirs in the wider Pacific Rim.

Purpose:

Provide the best-availale low-path AI data for the international Pacific Rim.

Time\_Period\_of\_Content:

Time\_Period\_Information:

Range\_of\_Dates/Times:

Beginning\_Date: Unknown  
Ending\_Date: Unknown

Currentness\_Reference:

Status:

Progress: Complete  
Maintenance\_and\_Update\_Frequency:

Spatial\_Domain:

Description\_of\_Geographic\_Extent: Japan, Vietnam, Mongolia, Russia, Alaska, US  
Bounding\_Coordinates:

West\_Bounding\_Coordinate:East\_Bounding\_Coordinate:North\_Bounding\_Coordinate:South\_Bounding\_Coordinate:

Keywords:

Theme:

Theme\_Keyword\_Thesaurus: None  
Theme\_Keyword: Avian Influenza database  
Theme\_Keyword: IRD Flubase  
Theme\_Keyword: low-path AI  
Theme\_Keyword: GIS modeling  
Theme\_Keyword: Model predictions

Place:

Place\_Keyword\_Thesaurus: None  
Place\_Keyword: Japan,  
Place\_Keyword: Vietnam,  
Place\_Keyword: Mongolia,  
Place\_Keyword: Russia,  
Place\_Keyword: Alaska U.S.

Taxonomy:

Keywords/Taxon:

Taxonomic\_Keyword\_Thesaurus: None  
Taxonomic\_Keywords: collection  
Taxonomic\_Keywords: multiple species  
Taxonomic\_Keywords: single species  
Taxonomic\_Keywords: animals  
Taxonomic\_Keywords: viruses

Taxonomic\_System:

Classification\_System/Authority:

Classification\_System\_Citation:

Citation\_Information:

Originator: Unknown  
Publication\_Date: Unknown  
Title:Geospatial\_Data\_Presentation\_Form:

Classification\_System\_Modifications: Follows authors and lab work and IRD/Flubase

Identification\_Reference:

Citation\_Information:

Originator: Follows authors and lab work and IRD/Flubase  
Publication\_Date: Unknown  
Title:Geospatial\_Data\_Presentation\_Form:

Taxonomic\_Procedures: Follows authors and lab work and IRD/Flubase  
Taxonomic\_Completeness: As per lab work by authors  
Vouchers:

Specimen: individuals  
Repository:

Contact\_Information:

Contact\_Person\_Primary:

Contact\_Person: Authors and their labs

Contact\_Address:

Address\_Type:City:State\_or\_Province:Postal\_Code:

Contact\_Voice\_Telephone:

Taxonomic\_Classification:

Taxon\_Rank\_Name:Taxon\_Rank\_Value:

Access\_Constraints: None  
Use\_Constraints: None  
Point\_of\_Contact:

Contact\_Information:

Contact\_Person\_Primary:

Contact\_Person:

Contact\_Address:

Address\_Type:Address:City:State\_or\_Province:Postal\_Code:

Contact\_Voice\_Telephone:

Data\_Set\_Credit: Authors and manuscript forthcoming, funded by eASIA  
Security\_Information:

Security\_Classification\_System: None, but ask authors for details  
Security\_Classification: Unclassified  
Security\_Handling\_Description: None, but ask authors for details

Native\_Data\_Set\_Environment: Labs from authors  
Analytical\_Tool:

Analytical\_Tool\_Description: Lab work by authors  
Tool\_Access\_Information:

Tool\_Access\_Instructions:

Data\_Quality\_Information:

Attribute\_Accuracy:

Attribute\_Accuracy\_Report: Correct as reported and based on lab work.

Logical\_Consistency\_Report: Consistent as per authors and lab  
Completeness\_Report: Complete as per authors and lab  
Positional\_Accuracy:

Horizontal\_Positional\_Accuracy:

Horizontal\_Positional\_Accuracy\_Report:

Accurate from GPS and place names.
5 decimals were used for decimal degrees, WGS84

Vertical\_Positional\_Accuracy:

Vertical\_Positional\_Accuracy\_Report: Meters,a s taken from GPS and GIS

Lineage:

Methodology:

Methodology\_Type: Field and lab  
Methodology\_Identifier:

Methodology\_Keyword\_Thesaurus: None  
Methodology\_Keyword: Field work  
Methodology\_Keyword: Lab work  
Methodology\_Keyword: Machine Learning  
Methodology\_Keyword: Data Mining  
Methodology\_Keyword: Predictions  
Methodology\_Keyword: Geographic Information Systems GIS

Methodology\_Description:

Methods described in a workflow etc in a manuscript by the authors

Methodology\_Citation:

Citation\_Information:

Originator: follows authors and as described in the manuscript.  
Originator: See here for a reference  
Originator:Originator:

Herrick, K.A., F. Huettmann and M. A Lindgren (2013) A global model of avian

Originator:

influenza prediction in wild birds: the importance of northern regions. Veterinary

Originator: Research 44(1): 42. doi: 10.1186/1297-9716-44-42.  
Originator:Publication\_Date: Unknown  
Title:Geospatial\_Data\_Presentation\_Form:

Process\_Step:

Process\_Description: No process steps have been described for this data set  
Process\_Date: Unknown

Spatial\_Data\_Organization\_Information:

Indirect\_Spatial\_Reference: Local location names  
Direct\_Spatial\_Reference\_Method: Raster

Spatial\_Reference\_Information:

Horizontal\_Coordinate\_System\_Definition:

Geographic:

Latitude\_Resolution: 0.00010000  
Longitude\_Resolution: 0.00100000  
Geographic\_Coordinate\_Units: Decimal degrees

Geodetic\_Model:

Horizontal\_Datum\_Name: World Geodetic System of 1984  
Ellipsoid\_Name: World Geodetic System of 1984  
Semi-major\_Axis: 6378137  
Denominator\_of\_Flattening\_Ratio: 298.25722210088

Entity\_and\_Attribute\_Information:

Detailed\_Description:

Entity\_Type:

Entity\_Type\_Label: PooledCleanbyFH5.csv  
Entity\_Type\_Definition: Comma Separated Value (CSV) file containing data.  
Entity\_Type\_Definition\_Source: Producer Defined

Attribute:

Attribute\_Label: FID  
Attribute\_Definition:Attribute\_Definition\_Source: Producer Defined  
Attribute\_Domain\_Values:

Range\_Domain:

Range\_Domain\_Minimum: 0  
Range\_Domain\_Maximum: 40835

Attribute:

Attribute\_Label: FHSampleID  
Attribute\_Definition:Attribute\_Definition\_Source: Producer Defined  
Attribute\_Domain\_Values:

Unrepresentable\_Domain:

Attribute:

Attribute\_Label: LapSampleI  
Attribute\_Definition:Attribute\_Definition\_Source: Producer Defined  
Attribute\_Domain\_Values:

Range\_Domain:

Range\_Domain\_Minimum: 0.0  
Range\_Domain\_Maximum: 1797.0

Attribute:

Attribute\_Label: Internal\_S  
Attribute\_Definition:Attribute\_Definition\_Source: Producer Defined  
Attribute\_Domain\_Values:

Unrepresentable\_Domain:

Attribute:

Attribute\_Label: Collection  
Attribute\_Definition:Attribute\_Definition\_Source: Producer Defined  
Attribute\_Domain\_Values:

Range\_Domain:

Range\_Domain\_Minimum: 0.0  
Range\_Domain\_Maximum: 31.0

Attribute:

Attribute\_Label: Collecti\_1  
Attribute\_Definition:Attribute\_Definition\_Source: Producer Defined  
Attribute\_Domain\_Values:

Range\_Domain:

Range\_Domain\_Minimum: 0.0  
Range\_Domain\_Maximum: 12.0

Attribute:

Attribute\_Label: Collecti\_2  
Attribute\_Definition:Attribute\_Definition\_Source: Producer Defined  
Attribute\_Domain\_Values:

Range\_Domain:

Range\_Domain\_Minimum: 2005.0  
Range\_Domain\_Maximum: 2018.0

Attribute:

Attribute\_Label: Collecti\_3  
Attribute\_Definition:Attribute\_Definition\_Source: Producer Defined  
Attribute\_Domain\_Values:

Unrepresentable\_Domain:

Attribute:

Attribute\_Label: Collecti\_4  
Attribute\_Definition:Attribute\_Definition\_Source: Producer Defined  
Attribute\_Domain\_Values:

Enumerated\_Domain:

Enumerated\_Domain\_Value:Enumerated\_Domain\_Value\_Definition:Enumerated\_Domain\_Value\_Definition\_Source: Producer defined

Attribute\_Domain\_Values:

Enumerated\_Domain:

Enumerated\_Domain\_Value: Alaska-USA  
Enumerated\_Domain\_Value\_Definition:Enumerated\_Domain\_Value\_Definition\_Source: Producer defined

Attribute\_Domain\_Values:

Enumerated\_Domain:

Enumerated\_Domain\_Value: Russia  
Enumerated\_Domain\_Value\_Definition:Enumerated\_Domain\_Value\_Definition\_Source: Producer defined

Attribute\_Domain\_Values:

Enumerated\_Domain:

Enumerated\_Domain\_Value: Mongolia  
Enumerated\_Domain\_Value\_Definition:Enumerated\_Domain\_Value\_Definition\_Source: Producer defined

Attribute\_Domain\_Values:

Enumerated\_Domain:

Enumerated\_Domain\_Value: Japan  
Enumerated\_Domain\_Value\_Definition:Enumerated\_Domain\_Value\_Definition\_Source: Producer defined

Attribute\_Domain\_Values:

Enumerated\_Domain:

Enumerated\_Domain\_Value:Enumerated\_Domain\_Value\_Definition:Enumerated\_Domain\_Value\_Definition\_Source: Producer defined

Attribute\_Domain\_Values:

Enumerated\_Domain:

Enumerated\_Domain\_Value: Vietnam  
Enumerated\_Domain\_Value\_Definition:Enumerated\_Domain\_Value\_Definition\_Source: Producer defined

Attribute:

Attribute\_Label: Latitude  
Attribute\_Definition:Attribute\_Definition\_Source: Producer Defined  
Attribute\_Domain\_Values:

Range\_Domain:

Range\_Domain\_Minimum: 9.09  
Range\_Domain\_Maximum: 71.29559999999998

Attribute:

Attribute\_Label: Longitude  
Attribute\_Definition:Attribute\_Definition\_Source: Producer Defined  
Attribute\_Domain\_Values:

Range\_Domain:

Range\_Domain\_Minimum: -179.08893  
Range\_Domain\_Maximum: 177.59668

Attribute:

Attribute\_Label: Hostspecie  
Attribute\_Definition:Attribute\_Definition\_Source: Producer Defined  
Attribute\_Domain\_Values:

Unrepresentable\_Domain:

Attribute:

Attribute\_Label: AIPresence  
Attribute\_Definition:Attribute\_Definition\_Source: Producer Defined  
Attribute\_Domain\_Values:

Enumerated\_Domain:

Enumerated\_Domain\_Value:Enumerated\_Domain\_Value\_Definition:Enumerated\_Domain\_Value\_Definition\_Source: Producer defined

Attribute\_Domain\_Values:

Enumerated\_Domain:

Enumerated\_Domain\_Value: Negative  
Enumerated\_Domain\_Value\_Definition:Enumerated\_Domain\_Value\_Definition\_Source: Producer defined

Attribute\_Domain\_Values:

Enumerated\_Domain:

Enumerated\_Domain\_Value: Positive  
Enumerated\_Domain\_Value\_Definition:Enumerated\_Domain\_Value\_Definition\_Source: Producer defined

Attribute\_Domain\_Values:

Enumerated\_Domain:

Enumerated\_Domain\_Value:Enumerated\_Domain\_Value\_Definition:Enumerated\_Domain\_Value\_Definition\_Source: Producer defined

Attribute\_Domain\_Values:

Enumerated\_Domain:

Enumerated\_Domain\_Value: Presence  
Enumerated\_Domain\_Value\_Definition:Enumerated\_Domain\_Value\_Definition\_Source: Producer defined

Attribute:

Attribute\_Label: Aisubtype  
Attribute\_Definition:Attribute\_Definition\_Source: Producer Defined  
Attribute\_Domain\_Values:

Unrepresentable\_Domain:

Attribute:

Attribute\_Label: Lab  
Attribute\_Definition:Attribute\_Definition\_Source: Producer Defined  
Attribute\_Domain\_Values:

Enumerated\_Domain:

Enumerated\_Domain\_Value: IRD U.S.  
Enumerated\_Domain\_Value\_Definition:Enumerated\_Domain\_Value\_Definition\_Source: Producer defined

Attribute\_Domain\_Values:

Enumerated\_Domain:

Enumerated\_Domain\_Value: Russia  
Enumerated\_Domain\_Value\_Definition:Enumerated\_Domain\_Value\_Definition\_Source: Producer defined

Attribute\_Domain\_Values:

Enumerated\_Domain:

Enumerated\_Domain\_Value: Japan  
Enumerated\_Domain\_Value\_Definition:Enumerated\_Domain\_Value\_Definition\_Source: Producer defined

Attribute:

Attribute\_Label: FHlowpath  
Attribute\_Definition:Attribute\_Definition\_Source: Producer Defined  
Attribute\_Domain\_Values:

Enumerated\_Domain:

Enumerated\_Domain\_Value: no  
Enumerated\_Domain\_Value\_Definition:Enumerated\_Domain\_Value\_Definition\_Source: Producer defined

Attribute\_Domain\_Values:

Enumerated\_Domain:

Enumerated\_Domain\_Value: yes  
Enumerated\_Domain\_Value\_Definition:Enumerated\_Domain\_Value\_Definition\_Source: Producer defined

Attribute:

Attribute\_Label: alt\_1  
Attribute\_Definition:Attribute\_Definition\_Source: Producer Defined  
Attribute\_Domain\_Values:

Enumerated\_Domain:

Enumerated\_Domain\_Value: -9999  
Enumerated\_Domain\_Value\_Definition: No Data  
Enumerated\_Domain\_Value\_Definition\_Source: Producer defined

Attribute\_Domain\_Values:

Range\_Domain:

Range\_Domain\_Minimum: -1  
Range\_Domain\_Maximum: 3613

Attribute:

Attribute\_Label: slope\_deg  
Attribute\_Definition:Attribute\_Definition\_Source: Producer Defined  
Attribute\_Domain\_Values:

Enumerated\_Domain:

Enumerated\_Domain\_Value: -9999  
Enumerated\_Domain\_Value\_Definition: No Data  
Enumerated\_Domain\_Value\_Definition\_Source: Producer defined

Attribute\_Domain\_Values:

Range\_Domain:

Range\_Domain\_Minimum: 0  
Range\_Domain\_Maximum: 5

Attribute:

Attribute\_Label: tmean\_34  
Attribute\_Definition:Attribute\_Definition\_Source: Producer Defined  
Attribute\_Domain\_Values:

Enumerated\_Domain:

Enumerated\_Domain\_Value: -9999  
Enumerated\_Domain\_Value\_Definition: No Data  
Enumerated\_Domain\_Value\_Definition\_Source: Producer defined

Attribute\_Domain\_Values:

Range\_Domain:

Range\_Domain\_Minimum: -279  
Range\_Domain\_Maximum: 279

Attribute:

Attribute\_Label: tmean\_6  
Attribute\_Definition:Attribute\_Definition\_Source: Producer Defined  
Attribute\_Domain\_Values:

Enumerated\_Domain:

Enumerated\_Domain\_Value: -9999  
Enumerated\_Domain\_Value\_Definition: No Data  
Enumerated\_Domain\_Value\_Definition\_Source: Producer defined

Attribute\_Domain\_Values:

Range\_Domain:

Range\_Domain\_Minimum: 14  
Range\_Domain\_Maximum: 298

Attribute:

Attribute\_Label: tmean\_9  
Attribute\_Definition:Attribute\_Definition\_Source: Producer Defined  
Attribute\_Domain\_Values:

Enumerated\_Domain:

Enumerated\_Domain\_Value: -9999  
Enumerated\_Domain\_Value\_Definition: No Data  
Enumerated\_Domain\_Value\_Definition\_Source: Producer defined

Attribute\_Domain\_Values:

Range\_Domain:

Range\_Domain\_Minimum: -6  
Range\_Domain\_Maximum: 280

Attribute:

Attribute\_Label: tmean\_12  
Attribute\_Definition:Attribute\_Definition\_Source: Producer Defined  
Attribute\_Domain\_Values:

Enumerated\_Domain:

Enumerated\_Domain\_Value: -9999  
Enumerated\_Domain\_Value\_Definition: No Data  
Enumerated\_Domain\_Value\_Definition\_Source: Producer defined

Attribute\_Domain\_Values:

Range\_Domain:

Range\_Domain\_Minimum: -319  
Range\_Domain\_Maximum: 263

Attribute:

Attribute\_Label: prox\_coast  
Attribute\_Definition:Attribute\_Definition\_Source: Producer Defined  
Attribute\_Domain\_Values:

Enumerated\_Domain:

Enumerated\_Domain\_Value: -9999  
Enumerated\_Domain\_Value\_Definition: No Data  
Enumerated\_Domain\_Value\_Definition\_Source: Producer defined

Attribute\_Domain\_Values:

Range\_Domain:

Range\_Domain\_Minimum: 0  
Range\_Domain\_Maximum: 19

Attribute:

Attribute\_Label: prox\_roads  
Attribute\_Definition:Attribute\_Definition\_Source: Producer Defined  
Attribute\_Domain\_Values:

Enumerated\_Domain:

Enumerated\_Domain\_Value: -9999  
Enumerated\_Domain\_Value\_Definition: No Data  
Enumerated\_Domain\_Value\_Definition\_Source: Producer defined

Attribute\_Domain\_Values:

Range\_Domain:

Range\_Domain\_Minimum: 0  
Range\_Domain\_Maximum: 5

Attribute:

Attribute\_Label: prox\_roa\_1  
Attribute\_Definition:Attribute\_Definition\_Source: Producer Defined  
Attribute\_Domain\_Values:

Enumerated\_Domain:

Enumerated\_Domain\_Value: -9999  
Enumerated\_Domain\_Value\_Definition: No Data  
Enumerated\_Domain\_Value\_Definition\_Source: Producer defined

Attribute\_Domain\_Values:

Range\_Domain:

Range\_Domain\_Minimum: 0  
Range\_Domain\_Maximum: 5

Attribute:

Attribute\_Label: prox\_coa\_1  
Attribute\_Definition:Attribute\_Definition\_Source: Producer Defined  
Attribute\_Domain\_Values:

Enumerated\_Domain:

Enumerated\_Domain\_Value: -9999  
Enumerated\_Domain\_Value\_Definition: No Data  
Enumerated\_Domain\_Value\_Definition\_Source: Producer defined

Attribute\_Domain\_Values:

Range\_Domain:

Range\_Domain\_Minimum: 0  
Range\_Domain\_Maximum: 19

Attribute:

Attribute\_Label: pigs  
Attribute\_Definition:Attribute\_Definition\_Source: Producer Defined  
Attribute\_Domain\_Values:

Enumerated\_Domain:

Enumerated\_Domain\_Value: -9999  
Enumerated\_Domain\_Value\_Definition: No Data  
Enumerated\_Domain\_Value\_Definition\_Source: Producer defined

Attribute\_Domain\_Values:

Range\_Domain:

Range\_Domain\_Minimum: 0  
Range\_Domain\_Maximum: 598

Attribute:

Attribute\_Label: poultry  
Attribute\_Definition:Attribute\_Definition\_Source: Producer Defined  
Attribute\_Domain\_Values:

Enumerated\_Domain:

Enumerated\_Domain\_Value: -9999  
Enumerated\_Domain\_Value\_Definition: No Data  
Enumerated\_Domain\_Value\_Definition\_Source: Producer defined

Attribute\_Domain\_Values:

Range\_Domain:

Range\_Domain\_Minimum: 0  
Range\_Domain\_Maximum: 4387

Attribute:

Attribute\_Label: kopgei  
Attribute\_Definition:Attribute\_Definition\_Source: Producer Defined  
Attribute\_Domain\_Values:

Enumerated\_Domain:

Enumerated\_Domain\_Value: -9999  
Enumerated\_Domain\_Value\_Definition: No Data  
Enumerated\_Domain\_Value\_Definition\_Source: Producer defined

Attribute\_Domain\_Values:

Range\_Domain:

Range\_Domain\_Minimum: 12  
Range\_Domain\_Maximum: 62

Attribute:

Attribute\_Label: nlp  
Attribute\_Definition:Attribute\_Definition\_Source: Producer Defined  
Attribute\_Domain\_Values:

Enumerated\_Domain:

Enumerated\_Domain\_Value: -9999  
Enumerated\_Domain\_Value\_Definition: No Data  
Enumerated\_Domain\_Value\_Definition\_Source: Producer defined

Attribute\_Domain\_Values:

Range\_Domain:

Range\_Domain\_Minimum: 10  
Range\_Domain\_Maximum: 255

Attribute:

Attribute\_Label: hf  
Attribute\_Definition:Attribute\_Definition\_Source: Producer Defined  
Attribute\_Domain\_Values:

Enumerated\_Domain:

Enumerated\_Domain\_Value: -9999  
Enumerated\_Domain\_Value\_Definition: No Data  
Enumerated\_Domain\_Value\_Definition\_Source: Producer defined

Attribute\_Domain\_Values:

Range\_Domain:

Range\_Domain\_Minimum: 0  
Range\_Domain\_Maximum: 100

Attribute:

Attribute\_Label: cyclone  
Attribute\_Definition:Attribute\_Definition\_Source: Producer Defined  
Attribute\_Domain\_Values:

Enumerated\_Domain:

Enumerated\_Domain\_Value: -9999  
Enumerated\_Domain\_Value\_Definition: No Data  
Enumerated\_Domain\_Value\_Definition\_Source: Producer defined

Attribute\_Domain\_Values:

Range\_Domain:

Range\_Domain\_Minimum: 1  
Range\_Domain\_Maximum: 9

Attribute:

Attribute\_Label: prec\_3  
Attribute\_Definition:Attribute\_Definition\_Source: Producer Defined  
Attribute\_Domain\_Values:

Enumerated\_Domain:

Enumerated\_Domain\_Value: -9999  
Enumerated\_Domain\_Value\_Definition: No Data  
Enumerated\_Domain\_Value\_Definition\_Source: Producer defined

Attribute\_Domain\_Values:

Range\_Domain:

Range\_Domain\_Minimum: 2  
Range\_Domain\_Maximum: 144

Attribute:

Attribute\_Label: prec\_6  
Attribute\_Definition:Attribute\_Definition\_Source: Producer Defined  
Attribute\_Domain\_Values:

Enumerated\_Domain:

Enumerated\_Domain\_Value: -9999  
Enumerated\_Domain\_Value\_Definition: No Data  
Enumerated\_Domain\_Value\_Definition\_Source: Producer defined

Attribute\_Domain\_Values:

Range\_Domain:

Range\_Domain\_Minimum: 7  
Range\_Domain\_Maximum: 295

Attribute:

Attribute\_Label: prec\_9  
Attribute\_Definition:Attribute\_Definition\_Source: Producer Defined  
Attribute\_Domain\_Values:

Enumerated\_Domain:

Enumerated\_Domain\_Value: -9999  
Enumerated\_Domain\_Value\_Definition: No Data  
Enumerated\_Domain\_Value\_Definition\_Source: Producer defined

Attribute\_Domain\_Values:

Range\_Domain:

Range\_Domain\_Minimum: 14  
Range\_Domain\_Maximum: 407

Attribute:

Attribute\_Label: prec\_12  
Attribute\_Definition:Attribute\_Definition\_Source: Producer Defined  
Attribute\_Domain\_Values:

Enumerated\_Domain:

Enumerated\_Domain\_Value: -9999  
Enumerated\_Domain\_Value\_Definition: No Data  
Enumerated\_Domain\_Value\_Definition\_Source: Producer defined

Attribute\_Domain\_Values:

Range\_Domain:

Range\_Domain\_Minimum: 1  
Range\_Domain\_Maximum: 330

Attribute:

Attribute\_Label: glc2000  
Attribute\_Definition:Attribute\_Definition\_Source: Producer Defined  
Attribute\_Domain\_Values:

Enumerated\_Domain:

Enumerated\_Domain\_Value: -9999  
Enumerated\_Domain\_Value\_Definition: No Data  
Enumerated\_Domain\_Value\_Definition\_Source: Producer defined

Attribute\_Domain\_Values:

Range\_Domain:

Range\_Domain\_Minimum: 2  
Range\_Domain\_Maximum: 22

Attribute:

Attribute\_Label: bio\_1  
Attribute\_Definition:Attribute\_Definition\_Source: Producer Defined  
Attribute\_Domain\_Values:

Enumerated\_Domain:

Enumerated\_Domain\_Value: -9999  
Enumerated\_Domain\_Value\_Definition: No Data  
Enumerated\_Domain\_Value\_Definition\_Source: Producer defined

Attribute\_Domain\_Values:

Range\_Domain:

Range\_Domain\_Minimum: -137  
Range\_Domain\_Maximum: 274

Attribute:

Attribute\_Label: bio\_2  
Attribute\_Definition:Attribute\_Definition\_Source: Producer Defined  
Attribute\_Domain\_Values:

Enumerated\_Domain:

Enumerated\_Domain\_Value: -9999  
Enumerated\_Domain\_Value\_Definition: No Data  
Enumerated\_Domain\_Value\_Definition\_Source: Producer defined

Attribute\_Domain\_Values:

Range\_Domain:

Range\_Domain\_Minimum: 38  
Range\_Domain\_Maximum: 150

Attribute:

Attribute\_Label: bio\_3  
Attribute\_Definition:Attribute\_Definition\_Source: Producer Defined  
Attribute\_Domain\_Values:

Enumerated\_Domain:

Enumerated\_Domain\_Value: -9999  
Enumerated\_Domain\_Value\_Definition: No Data  
Enumerated\_Domain\_Value\_Definition\_Source: Producer defined

Attribute\_Domain\_Values:

Range\_Domain:

Range\_Domain\_Minimum: 14  
Range\_Domain\_Maximum: 68

Attribute:

Attribute\_Label: bio\_4  
Attribute\_Definition:Attribute\_Definition\_Source: Producer Defined  
Attribute\_Domain\_Values:

Enumerated\_Domain:

Enumerated\_Domain\_Value: -9999  
Enumerated\_Domain\_Value\_Definition: No Data  
Enumerated\_Domain\_Value\_Definition\_Source: Producer defined

Attribute\_Domain\_Values:

Range\_Domain:

Range\_Domain\_Minimum: 686  
Range\_Domain\_Maximum: 17370

Overview\_Description:

Entity\_and\_Attribute\_Overview:

Avian Infuenza Clean data set used for GIS model work with GIS overlays

Entity\_and\_Attribute\_Detail\_Citation: Huettmann et al. unpublished

Distribution\_Information:

Distributor:

Contact\_Information:

Contact\_Person\_Primary:

Contact\_Person: see authors

Contact\_Address:

Address\_Type:Address:City:State\_or\_Province:Postal\_Code:

Contact\_Voice\_Telephone:

Resource\_Description:

Digital dataset on low-path AI for the Pacific Rim in an ASCII format for GIS use

Distribution\_Liability: None  
Custom\_Order\_Process: Contact authors in person, by phone or email or mail  
Technical\_Prerequisites: PC IBM and WW

Metadata\_Reference\_Information:

Metadata\_Date: 20191105  
Metadata\_Contact:

Contact\_Information:

Contact\_Person\_Primary:

Contact\_Person: Falk Huettmann

Contact\_Address:

Address\_Type: mailing and physical  
Address: 419 Irving I  
City: Fairbanks  
State\_or\_Province: Alaska  
Postal\_Code: 99775  
Country: USA

Contact\_Voice\_Telephone:Contact\_Facsimile\_Telephone: 907 474 7882  
Contact\_Electronic\_Mail\_Address: fhuettmann@alaska.edu

Metadata\_Standard\_Name:

FGDC Biological Data Profile of the Content Standard for Digital Geospatial Metadata

Metadata\_Standard\_Version: FGDC-STD-001.1-1999  
Metadata\_Access\_Constraints: None  
Metadata\_Use\_Constraints: None  
Metadata\_Security\_Information:

Metadata\_Security\_Classification\_System: None  
Metadata\_Security\_Classification: Unclassified  
Metadata\_Security\_Handling\_Description: None

---

Generated by mp version 2.9.50 on Fri Jun 05 17:04:26 2020
